# Risk perceptions, attitudes, and knowledge of chikungunya among the public and health professionals: a systematic review

**DOI:** 10.1186/s41182-017-0061-x

**Published:** 2017-09-04

**Authors:** Tricia Corrin, Lisa Waddell, Judy Greig, Ian Young, Catherine Hierlihy, Mariola Mascarenhas

**Affiliations:** 1 Public Health Risk Sciences Division, National Microbiology Laboratory, Public Health Agency of Canada, Guelph, ON Canada; 2 Department of Population Medicine, University of Guelph, Guelph, ON Canada; 3 School of Occupational and Public Health, Ryerson University, Toronto, ON Canada

**Keywords:** Chikungunya, Systematic review, Attitudes, Knowledge, Perceptions

## Abstract

**Background:**

Recently, attention to chikungunya has increased due to its spread into previously non-endemic areas. Since there is no available treatment or vaccine, most intervention strategies focus on mosquito bite prevention and mosquito control, which require community involvement to be successful. Thus, our objective was to systematically review the global primary literature on the risk perceptions, attitudes, and knowledge of chikungunya among the public and health professionals to inform future research and improve our understanding on which intervention strategies are likely to be successful.

**Methods:**

Potentially relevant articles were identified through a standardized systematic review (SR) process consisting of the following steps: comprehensive search strategy in seven databases (Scopus, PubMed, CINAHL, CAB, LILACS, Agricola, and Cochrane) and a grey literature search of public health organizations, relevance screening, risk of bias assessment, and data extraction. Two independent reviewers performed each step. Reporting of this SR follows PRISMA reporting guidelines.

**Results:**

Thirty-seven relevant articles were identified. The majority of the articles were published since 2011 (83.8%) and reported on studies conducted in Asia (48.7%) and the Indian Ocean Islands (24.3%). The results were separated into four categories: general knowledge and perceptions on chikungunya; perceptions on the risk and severity of chikungunya; knowledge of chikungunya-harboring vectors and transmission; and knowledge, perceptions, and attitudes on mitigation practices. Overall, the systematic review found that risk perceptions, attitudes, and knowledge of chikungunya among the public and health professionals vary across populations and countries and knowledge is higher in areas that have experienced an outbreak.

**Conclusion:**

The results suggest that most of the affected populations in this study do not understand mosquito borne diseases or chikungunya and are therefore less likely to protect themselves from mosquito bites. While more research is required to improve the generalizability of this dataset, it appears that a lack of knowledge is an important barrier for motivating community level interventions and personal protection against mosquitoes.

**Electronic supplementary material:**

The online version of this article (doi:10.1186/s41182-017-0061-x) contains supplementary material, which is available to authorized users.

## Background

Chikungunya is an alphavirus that is transmitted to humans through mosquito bites. It causes a non-specific illness including high fever, severe joint pain, muscle pain, headache, nausea, fatigue, and rash in infected individuals [1, 2]. While most people recover from the acute illness in 1–2 weeks, there are a proportion of individuals that continue to suffer from chronic joint pain which can persist for weeks to years following infection [1, 2].

Historically, chikungunya virus (CHIKV) has circulated in Africa, Asia, and the Indian and Pacific Ocean Islands [2]. In 2013, the virus spread to the Americas and caused outbreaks in countries that harbor the vectors, *Aedes aegypti* and *Aedes albopictus* [2–4]. Cases of infected travelers in Europe and North America returning from CHIKV-affected countries have been documented as well as several small outbreaks in Europe due to importation of the virus into an area with suitable vectors [3, 5].

Chikungunya is an important public health concern as the virus continues to emerge into previously non-endemic areas such as the Americas, which have reported more than 1.7 million suspected or confirmed cases since 2013 [6]. In the USA, chikungunya has been a notifiable disease since 2015, and in the same year, the Centers for Disease Control and Prevention reported 679 travel-related cases of chikungunya from 44 states [3]. Canada has reported several hundred travel-related cases of chikungunya since it spread to the Americas [7].

Most intervention strategies have focused on mosquito control and mosquito bite prevention as there is currently no treatment or vaccine for CHIKV infection in humans [8]. The success of these intervention strategies relies on social factors such as knowledge, attitudes, and perceptions of the disease. It is important to understand how affected populations understand and perceive chikungunya, its transmission cycle, and the importance of control measures to determine what prevention strategies are likely to be successful. In addition, how and why the target population chooses to take preventative action against mosquito borne diseases like CHIKV is necessary to inform future education and control strategies. Thus, a systematic review was conducted to identify, assess, and analyze the global evidence on the knowledge, attitudes, and perceptions of CHIKV and its transmission in affected populations.

## Methods

### Research question, team, and protocol

This systematic review was conducted following internationally recognized procedures and is reported in accordance with the PRISMA guidelines [9–11].

The systematic review question is “what are risk perceptions, attitudes and/or knowledge of chikungunya among the public and health professionals?” A multidisciplinary team with expertise in knowledge synthesis, epidemiology, risk assessment, public health, and information science conducted the review.

Prior to the systematic review, a pre-specified systematic review protocol was developed which included the research question, definitions, inclusion criteria for relevance screening, risk of bias tool, and data extraction forms. The systematic review protocol and citation list of relevant articles is available in supplementary material (Additional files 1 and 2), and the dataset resulting from this review is available upon request.

### Search strategy and eligibility criteria

A scoping review of the global literature on chikungunya, conducted at the Public Health Agency of Canada (personal communication M. Mascarenhas 2017), served as the starting point for this systematic review. Briefly, the scoping review aimed to identify all relevant research on chikungunya; a search was conducted to capture all primary research in English, French, Spanish, or Portuguese. Seven electronic sources were selected based on their relevance to the scoping review. These were accessed through the Public Health Agency of Canada Library and included Scopus, PubMed, The Cumulative Index to Nursing and Allied Health Literature (CINAHL), CAB, LILACS (South America), Agricola, and the Cochrane Library for any relevant trials in the trial registry. The initial search was conducted on May 27, 2015, using a pre-tested search algorithm (Chikungunya OR CHIK OR CHIKV) OR (alphavirus AND mosquito* AND control). An updated search using the same electronic sources and algorithms was completed on January 6, 2017. A grey literature search of pre-specified public health organization (*n* = 19, list available upon request) websites was undertaken to identify any non-peer-reviewed studies or surveillance data that was not captured in the electronic search. All studies on any aspect of chikungunya or CHIKV were included and characterized. One of the scoping review categories was on studies describing “Public and health professionals/physicians’ knowledge, attitudes and/or risk perceptions towards chikungunya and potential prevention and control strategies.” The 45 studies from this category were considered for inclusion in this systematic review. Further details on the scoping review protocol and methods are available upon request.

### Relevance confirmation, risk of bias assessment, and data extraction

To ensure that the studies from the scoping review were applicable to the research question, a single relevance question appeared at the beginning of the risk of bias assessment and data extraction form to allow the reviewer to eliminate any irrelevant studies.

All studies were evaluated for their risk of bias using a pre-designed risk of bias assessment form (Additional file 1). The form was created to address both qualitative (8 criteria) and quantitative (11 criteria) studies. Previously designed critical appraisal tools for qualitative and quantitative studies were used to create this risk of bias form [12–14]. Each study received an overall risk of bias score where studies conducted to minimize bias in the results were assigned a low risk of bias ranking. If one or more criteria could not be assessed due to lack of reporting, an unclear risk of bias was appointed. Studies received a high risk of bias if one or more criteria were not met. A data extraction form was used to extract relevant information and results from each quantitative study. The extraction form included 9 questions designed to extract information on study design, demographics, and the results of the studies that fell into the following categories: perceptions about the severity of chikungunya disease; knowledge, perceptions, and attitudes on mitigation practices; knowledge on chikungunya; and knowledge on CHIKV harboring vectors and how CHIKV is transmitted. Outcomes more generally related knowledge, perceptions, and attitudes on mosquito borne diseases (MBDs), and mosquito control reported in relevant papers on chikungunya and chikungunya-affected populations were also captured to examine if general attitudes, perceptions, and knowledge were more closely correlated to knowledge, attitudes, and perceptions on the use of personal protective measures than knowledge on chikungunya or CHIKV specifically.

Both forms were pre-tested by all team members to ensure clarity of the questions, extraction of the right information to address our research question, and to ensure process consistency. Once pre-testing was completed, two reviewers extracted the data and evaluated the risk of bias for each paper independently. During both stages, conflicts between reviewers were resolved by consensus.

### Review management and data analysis

The scoping review steps, data extraction, and risk of bias assessment were conducted using the web-based systematic review software DistillerSR (Evidence Partners, Ottawa, Canada). The data was then exported to Microsoft Excel (Microsoft Corporation 2010) for descriptive analysis. Results with reference to “*n*” refer to the total number of samples, subjects, or participants for the presented outcome.

Meta-analysis models were developed using the statistical software STATA13 (StatCorp 2015). The metaprop package was used to obtain weighted average prevalence estimates for two outcomes: the proportion of the general public sample population that were aware of chikungunya in outbreak and non-outbreak populations and the proportion of the sample population that had knowledge on mosquito transmission of CHIKV by country. Based on the assumption that the prevalence estimates would have some heterogeneity between study populations, a random effects meta-analysis was conducted using the DerSimonian and Laird method [15]. In some cases, more than one observation per study was included in the meta-analysis. We did not account for the potential similarity of these results as they were all independent observations on different sampling frames. We evaluated how much heterogeneity between studies was not explained by random error using the value *I* ^2^ [16]. High heterogeneity, *I* ^2^ > 60%, was expected, and our goal was to investigate whether there were study level variables that explained the heterogeneity and to provide a graphic of the studies for the reader. We caution readers not to use the summary estimates as an estimate of the average outcome across studies given that estimates were obtained from very different populations and no study level variables explained all the heterogeneity between studies. In the forest plots, *p* = 0.00 is actually *p* < 0.01. This is an output of STATA13 (StatCorp 2015) and is not open for the user to alter or redefine.

Qualitative research studies were synthesized using a narrative review approach [17]. This included two reviewers independently reviewing the results of each study and descriptively summarizing the key results as reported by the study authors. Summaries from both reviewers were discussed and consolidated to arrive at the final narrative description. Only two relevant qualitative studies were identified in this review; therefore, we decided not to use a formal coding procedure in the analysis or develop interpretive across-study themes.

## Results

### Systematic review descriptive statistics

**Fig. 1:**
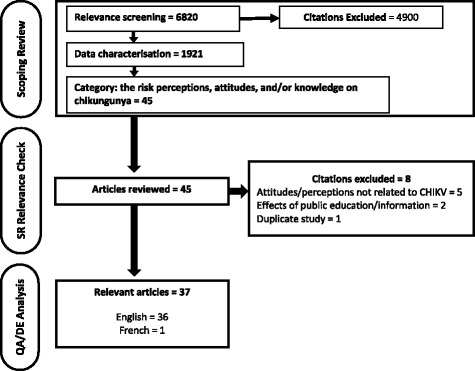
PRISMA flow diagram of articles through the systematic review process

There were 6820 citations screened for relevance in the scoping review project, of which 1921 studies were considered to be relevant primary research on chikungunya (Additional file 3). Only 45 of these were categorized to address knowledge, perceptions, and attitudes toward chikungunya and potential prevention and control strategies. Eight of these studies were deemed irrelevant during relevance confirmation, resulting in 37 total articles included in this systematic review. The flow of information through the systematic review process is depicted in Fig. 1.

All studies were published between 2007 and 2016, with 83.8% (*n* = 37) published since 2011. The majority of the articles (73.0%) reported on studies conducted in Asia (48.7%) and the Indian Ocean Islands (24.3%). Specifically, most of the research originated from India (41.0%) and La Réunion (18.0%), as shown in Table 1. The most widely used study design was cross-sectional (75.7%), followed by quasi-experiment (13.5%), qualitative (5.4%), case-control (2.7%), and longitudinal (2.7%). The main population studied was the general public (86.5%), and only a few studies had data collection processes that were informed by established theories of behavior change (24.3%).

**Table 1:** General characteristics of 37 included primary research publications

Category		Count (percentage)
Continent/country^a,b^		
Asia	India	16 (41.0%)
	Singapore	1 (2.6%)
	Sri Lanka	1 (2.6%)
Europe	France	2 (5.1%)
	Italy	1 (2.6%)
	Spain	1 (2.6%)
Indian Ocean Islands	La Réunion	7 (18.0%)
	Mauritius	2 (5.1%)
	Mayotte	2 (5.1%)
Americas	USA	2 (5.1%)
	Colombia	1 (2.6%)
	French Guiana	1 (2.6%)
	Nicaragua	1 (2.6%)
	US Virgin Islands	1 (2.6%)
Language		
	English	36 (97.3%)
	French	1 (2.7%)
Date of publication		
	2007–2010	6 (16.2%)
	2011–2016	31 (83.8%)
Risk of bias assessment^c^		
	Low risk of bias	16 (45.7%)
	Unclear risk of bias	16 (45.7%)
	High risk of bias	3 (8.6%)
Study design		
	Cross-sectional	28 (75.7%)
	Quasi-experiment	5 (13.5%)
	Qualitative	2 (5.4%)
	Case-control	1 (2.7%)
	Longitudinal	1 (2.7%)
Population		
	General public	32 (86.5%)
	Health professionals	5 (13.5%)
Theory of behavior change used		
	None	28 (75.7%)
	Health belief model	3 (8.1%)
	Stages of change theory	2 (5.4%)
	Theory of planned behavior	4 (10.8%)

^a^Total number sums to >37 as studies can fall into more than one category

^b^Total percentages do not equal 100 due to rounding

^c^Total number sums to 35 as qualitative studies were not given an overall risk of bias score

The 35 quantitative studies were ranked as having a low (45.7%), unclear (45.7%), or high (8.6%) overall risk of bias. The most common reason for an unclear risk of bias score was due to a lack of reporting on potential confounders (56.3%, *n* = 16) and a lack of clarity on whether tools to measure outcomes (e.g., questionnaires) were reliably tested and validated (81.3%, *n* = 16). For the two qualitative studies, the main quality assessment deficiencies were that the method of analysis, research design, and data collection were not clearly described in either study.

### General knowledge and perceptions on chikungunya

**Fig. 2:**
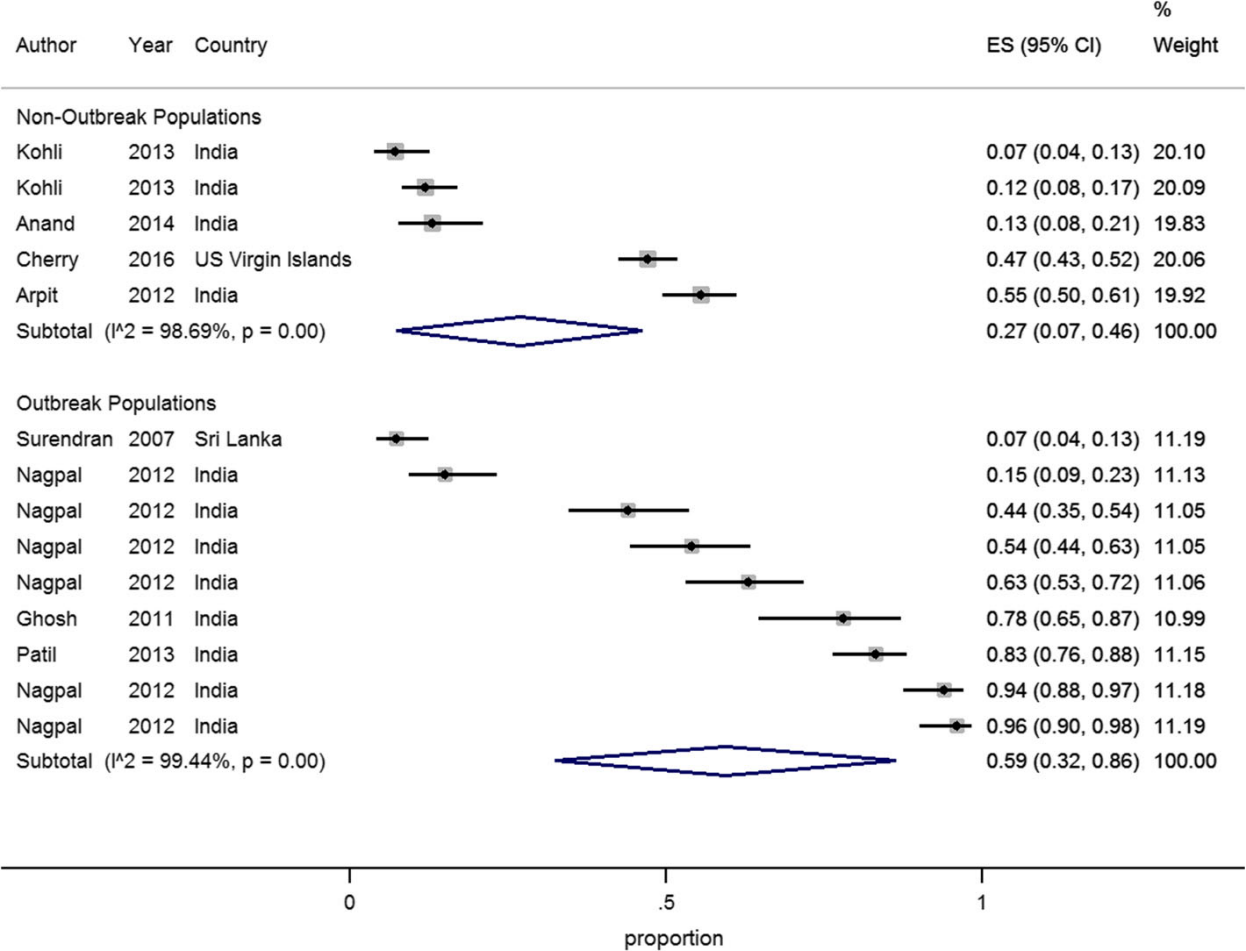
Meta-analysis of the proportion (ES, 95% CI) of the general public sample population that were aware of chikungunya in outbreak and non-outbreak populations

Awareness of chikungunya was evaluated in nine studies. Eight studies conducted in Asia (*n* = 7) and the Caribbean (*n* = 1) dichotomized results and reported awareness of chikungunya among the general public, which varied from 7–96% as shown in Fig. 2 [18–25]. The meta-analysis in Fig. 2 shows that awareness of chikungunya was highest in the four studies where an outbreak was on-going [19, 22, 23] and among urban link workers who are responsible for implementing anti-larval measures through door-to-door visits in their community [21]. In contrast, a study conducted in Sri Lanka where an outbreak was occurring only reported an awareness rate of 7% [24]. However, it is unclear from the study if chikungunya was an emerging disease and whether any previous public education had occurred.

Before traveling from the USA to the Dominican Republic, 19% of community service volunteers (*n* = 102) reported they had knowledge of chikungunya [26]. Of these volunteers, 87% (*n* = 102) had visited a health-care provider for a pre-travel consultation [26]. In Nicaragua, 93.5% of individuals (*n* = 848) from a general population sample considered themselves informed about chikungunya, but actual knowledge was not evaluated [27]. Whereas in a study in India, only 8.9% of the surveyed population (*n* = 740) had correct knowledge about the virus etiology [28]. When students in French Guiana were asked about the duration of chikungunya, 51% (*n* = 1462) answered correctly 3 weeks to several months [29].

Three studies evaluated general knowledge of mosquito borne diseases [30–32]. One of the studies attempted to gauge the level of understanding about mosquito borne diseases in the population by sampling 1506 individuals and having them rank their knowledge on a scale of 0–10, with 0 being “do not understand at all” and 10 being “understand completely” [30]. The mean score was 5.6 (SD 2.85), indicating some knowledge, but a lack of in-depth understanding [30]. General knowledge on mosquito borne diseases in India among a group of health-care workers was 83% (*n* = 100) [31] whereas in a different study 88.1% (*n* = 119) [32] of the general population sample was considered to have general knowledge.

Gender was shown to be a significant predictor of knowledge of chikungunya as a mosquito borne disease in two studies from India [20, 33]. In both studies, females were shown to have more knowledge about mosquito borne disease (13.8%, *n* = 350) compared to males (4.8%, *n* = 350), *p* value 0.01 [20] and chikungunya (OR 1.37; 95% CI 1.11–1.71; *p* = 0.003; *n* = 1674) [33]. Other significant socio-economic factors included less knowledge among illiterate participants (OR 0.65; 95% CI: 0.51–0.82); *p* < 0.001) and participants over the age of 30 (OR 0.67; 95% CI: 0.54–0.83; *p* < 0.01) compared to those that were literate or between the age of 18–30 [33].

Eight studies measured knowledge of chikungunya [22, 30, 31, 34, 35] and mosquito borne disease [29, 36, 37] symptoms among populations affected by the disease. Knowledge of the signs and symptoms of chikungunya varied across different study populations; however, joint pain, fever, and swelling were commonly identified [22, 30, 35]. In a group of health-care workers in India, 22% (*n* = 100) had knowledge on chikungunya symptoms [31]. The results of a case-control study where the cases were diagnosed with chikungunya showed that those with first-hand experience of the disease were more aware of the symptoms of the disease in comparison with the non-cases [35]. In a study of health-care workers and medical students in two Colombian cities, participants correctly identified polyarthralgia and fever as the most frequent symptoms of chikungunya: 91.9% in Pereira (*n* = 99) and 86.9% in Cartagena (*n* = 107) [34]. For mosquito borne diseases, the most frequently reported symptoms included limb swelling, fever, headache, myalgia, arthralgia, and skin rashes [29, 36]. In one study, knowledge about the symptoms of mosquito borne disease varied depending on whether larval breeding sites were identified around the home [37]. In the absence of larval breeding sites on an individual’s property, 37.5% surveyed (*n* = 160) had no knowledge of the symptoms of mosquito borne diseases compared to 62.5% of individuals where larval breeding sites were identified on their property [37]. Larval breeding sites around homes was used as a surrogate measure for risk of exposure, and knowledge of symptoms is presumably due to personal experience with someone in the home being ill from CHIKV.

Perceptions on the treatment of chikungunya were addressed in four studies, two of which sampled populations of health professionals [26, 30, 38, 39]. A group of final year medical students (*n* = 314) from Singapore were surveyed and 20.1% believed that it is necessary to isolate patients who have chikungunya [38]. Five doctors (100%) surveyed in India believed that the traditional system of medicine called Ayurveda was more effective than other systems of medicine to treat chikungunya [39]. In addition, these same doctors all believed that homeopathic medicine can cure chikungunya completely, but only 60% were aware of the efficacy and adverse effects of the treatment used [39]. In France, 1506 participants from the general public gave a mean score of 7.01 (SD 2.24) out of 10 that treatment can help with mosquito borne diseases including chikungunya, where 0 indicates no effect of treatment and 10 indicates treatment is highly effective [30]. From a group of community service volunteers in the USA with knowledge on chikungunya, 68% (*n* = 19) believed there was no available treatment for the disease [26].

### Perceptions on the risk and severity of chikungunya

Six studies looked at perceptions of the risk and severity of chikungunya among populations of the general public that were recently affected by an outbreak (Table 2) [26, 29, 40–43]. The perceived severity of the disease was deemed to be high or moderate by 20.5–93.4% of the participants across three studies (*n* = 1021, *n* = 880) conducted on the Indian Ocean Islands [41, 43] and 94.5% (*n* = 18) in a group of USA community volunteers traveling to the Dominican Republic [26]. Following a small CHIKV outbreak in Italy, 49.8% (*n* = 293) of the population in the affected region perceived a high risk associated with chikungunya, and 83.2% (*n* = 291) were worried about the disease in the near future [40]. Whereas a study conducted in France (*n* = 1506) reported that the perceived severity of mosquito borne diseases was moderate, mean score of 7.07 (SD 1.94), and they were less concerned about the risk of contracting a mosquito borne disease, mean score 4.89 (SD 3.15), which may be because many mosquito borne diseases are not endemic in France [30]. Another study conducted in the USA reported that 63% of the surveyed population (*n* = 87) were a little or very worried about contracting a mosquito borne disease such as chikungunya or dengue, but only 15% (*n* = 88) believed it was likely that someone they knew would contract a mosquito borne disease [44].

**Table 2:** Perceived risk and severity of chikungunya and mosquito borne diseases (MBDs) among the general public

REF	Author (year)	Location	Sample size	Proportion or mean score	Description of outcome
Chikungunya					
[29]	Fritzell (2016)	French Guiana	1462	Mean score = 5.37 95% CI: 5.12–5.63	Perceived risk of exposure to chikungunya
[40]	Moro (2010)	Italy	293	49.8%	Perceived a high risk associated with chikungunya
[40]	Moro (2010)	Italy	291	83.2%	Worried about chikungunya in the near future
[41]	Setbon (2008)	La Réunion	1021	2.7% (score: 0–3) 20.5% (score: 4–6) 76.8% (score: 7–10)	Perceived severity of chikungunya on a scale of 0–10 (0 - low, 10 - high)
[42]	Thuilliez (2014)	La Réunion	1024	47.7%	Perceived risk of a new chikungunya outbreak was reasonable or high
[43]	Raude (2009)	Mayotte	880	93.4% (high or moderate) 6.6% (low or none)	Perceived severity of chikungunya
[26]	Millman (2016)	USA	18	94.5%	Perceived possible risk of exposure in Dominican Republic
Mosquito borne diseases (MBD)					
[30]	Raude (2012)	France	1506	Mean score = 7.07 SD = 1.94	Perception on how serious MBDs are on a scale of 0–10 (0 – not serious, 10 – serious)
[30]	Raude (2012)	France	1506	Mean score = 4.89 SD = 3.15	Worried about the risk of contracting MBDs on a scale of 0–10 (0 – not worried at all, 10 – extremely worried)
[33]	Boratne (2010)	India	1674	54.9%	Perceived MBDs as a serious problem in the area (54.9%)
[24]	Surendran (2007)	Sri Lanka	162	29% (severe) 71% (moderate)	Perception of the mosquito problem
[61]	Boyer (2014)	La Réunion	Not reported	78.9%	Good knowledge of vectorial risk
[25]	Cherry (2016)	US Virgin Islands	443	43% (not concerned) 49.5% (mildly concerned) 7.5% (very concerned)	Concerned about getting a MBD during their trip
[44]	Adalja (2016)	USA	87	37% (had not thought about it or were not worried at all) 63% (a little worried or very worried)	Level of worry about MBDs like dengue or chikungunya
[44]	Adalja (2016)	USA	88	85% (very unlikely, unlikely, or uncertain) 15% (likely or very likely)	Perceived likelihood that someone they know could contract dengue or chikungunya while living in their community

*MBD* mosquito borne disease, *SD* Standard deviation, 95% CI = 95% confidence interval

### Knowledge of chikungunya-harboring vectors and transmission

**Fig. 3:**
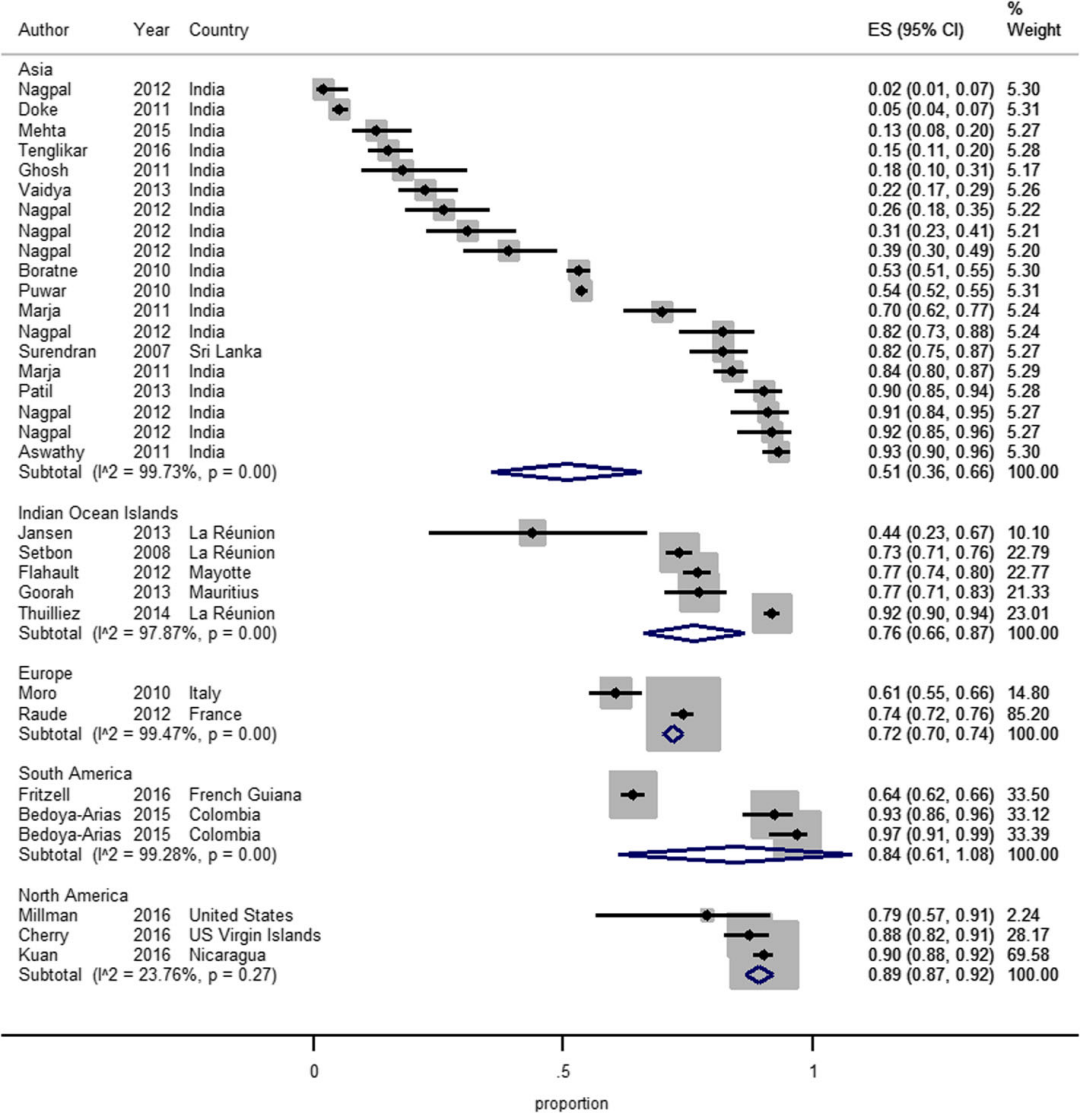
Meta-analysis of the proportion (ES, 95% CI) of the sample population that had knowledge on mosquito transmission of CHIKV by country

Twenty-four studies assessed what proportion of a study population knew that CHIKV is transmitted to humans through mosquito bites [19, 22–30, 32–36, 40–42, 45–50]. All of these studies looked at the general population with the exception of one study that looked at a group of health professionals in Colombia. The results of the meta-analysis in Fig. 3 indicate that knowledge levels on transmission of CHIKV by mosquitoes range widely between 2 and 97% across studies.

A few studies of the general public conducted on the Indian Ocean Islands found that there are misconceptions on how and why CHIKV is transmitted. One study (*n* = 1035) reported 73.3% (*n* = 989) of participants were aware that mosquitoes transmit CHIKV; however, participants also believed that CHIKV could be transmitted through the air (36.5%, *n* = 996) and/or direct human contact (33.9%, *n* = 999) [41]. When asked how CHIKV was introduced to the island, responses varied from the bodies of tsunami victims that reached the island (33.1%, *n* = 983), crews of quarantined ships (61.7%, *n* = 981) to intentional introduction by secret agents (26.8%, *n* = 965) [41]. A large proportion of a population in Mayotte (*n* = 888) believed that the proximate cause of CHIKV is mosquitoes (77%, *n* = 835), but the ultimate cause is God’s punishment (55.4%, *n* = 802), migrants (36.8%, *n* = 742), and witchcraft/sorcery (12.5%, *n* = 807) [43]. This same group of participants (*n* = 888) also believed the virus can be transmitted by blood transfusion (64%), sexual intercourse (40%), animals (30%), and by shaking the hand of an infected person (21%) [48].

Knowledge of the general public on mosquito breeding sites was assessed in ten studies [18, 20, 23, 31–33, 35, 36, 45, 51]. With the exception of one study from Spain, all were conducted in India. Between 2008 and 2010, 84.9% of a studied population in Spain (*n* = 820) knew about the larval habitats of tiger mosquitoes [51]. In India, 89% of a surveyed group of health-care workers (*n* = 100) had knowledge of mosquito breeding sites [31]. Across all studies (Table 3), stagnant water (29–85%) [18, 20, 23, 33, 36] and coconut shells (4.2–73%) [20, 33, 35, 45] were most commonly identified as places where mosquitoes breed. Other breeding places such as cement baths [35], vehicle tires [20, 33], water storage jars [35], broken utensils [45], cracks in the wall, drain and polluted water [32], and desert coolers [20] were cited with lower frequency. Those with first-hand experience of the disease in a case-control study conducted in India were found to have less knowledge on mosquito breeding sites than those who were selected as non-cases [35]. For example, 73% of individuals who did not have the disease (*n* = 450) knew that mosquitoes breed in coconut shells as opposed to 35% of those who had contracted chikungunya (*n* = 150) [35].

**Table 3:** General public’s knowledge on mosquito breeding sites

REF	Author (year)	Location	Sample size	Results
Stagnant water				
[18]	Anand (2014)	India	100	Stagnant polluted water (29%), stagnant clean water (68%)
[33]	Boratne (2010)	India	1674	Stagnant water (60.7%), ditches (35%), ponds (24%)
[20]	Kohli (2013)	India	350	Stagnant water (60.9%), blocked drains (40%)
[23]	Ghosh (2011)	India	50	Stagnant water (66%)
[36]	Tenglikar (2016)	India	247	Dirty stagnant water (85%)
[32]	Mehta (2015)	India	119	Drain and polluted water (58.8%)
Water - storage and other				
[35]	Majra (2011)	India	150 cases, 450 non-cases	Water storage jars—cases (48%), non-cases (66%)
[32]	Mehta (2015)	India	119	Clean water collection (27.7%)
[36]	Tenglikar (2016)	India	247	Artificial collection of water/water storage (14.6%)
Small containers				
[18]	Anand (2014)	India	100	Desert coolers (20%)
[45]	Aswathy (2011)	India	300	Coconut shells and broken utensils (69%)
[33]	Boratne (2010)	India	1674	Vehicle tires (2.6%); coconut shells (4.2%)
[20]	Kohli (2013)	India	350	Old tires, broken pots and coconut shells (41.4%), desert coolers (26.3%)
[35]	Majra (2011)	India	150 cases, 450 non-cases	Coconut shells—cases (35%), non-cases (73%), tires—cases (13%), non-cases (54%)
Other				
[23]	Ghosh (2011)	India	50	Cracks in walls (2%), earth and air (2%)
[35]	Majra (2011)	India	150 cases, 450 non-cases	Cement baths—cases (28%), non-cases (66%)

### Knowledge, perceptions, and attitudes on mitigation practices

The perception of personal control or “self-responsibility” for mitigation of CHIKV varied among four studies of the general public. In a study conducted in India, 42.7% of participants (*n* = 178) believed that they were personally responsible for the mitigation of CHIKV [46]. On the Indian Ocean Islands where a large chikungunya outbreak was occurring for the first time, 54% (*n* = 1021) [41] did not believe they had any control over the disease whereas another study on the island reported that 83.9% (*n* = 888) [43] believed that chikungunya is controllable. In France, participants indicated they have a moderate level of control over their risk of contracting mosquito borne diseases, mean 7.12 (SD 2.39) on a scale of 0–10, with 0 having absolutely no control and 10 having an extreme amount of control [30].

Knowledge on government measures for the prevention and control of mosquito borne diseases varied across three studies conducted in India, 21.1% (*n* = 1674), 84% (*n* = 100), and 88.9% (*n* = 135) [18, 32, 33]. Following an outbreak in La Réunion, 55% (*n* = 999) of the study participants from the general public believed that public authorities had done everything in their power to stop the spread of chikungunya [41]. However, during the same outbreak, the management directives from health authorities in La Réunion were perceived as ineffective by 60.4% (*n* = 91) of health professionals [52]. Variation on the perceived effectiveness of mitigation measures was also seen between studies of the general public. The perceived effectiveness of CHIKV protective measures was positive in Mayotte (79.7%, *n* = 888) and La Réunion (64.8%, *n* = 1024) [42, 43], but in India, only 14% (*n* = 84) and 28.3% (*n* = 120) of participants from two studies believed the protective measures to be adequate [18, 32]. The participants from one of the studies in India cited corruption, a late reactive approach, and a lack of accountability in the system as reasons for the inadequacy and ineffective mitigation [18].

Thirteen studies [18–23, 31–33, 35, 43, 45, 46] captured knowledge of the general public on vector control and personal protective measures. The majority (92.3%, *n* = 13) of these studies were conducted in India. Table 4 shows many differences between knowledge of various protective measures across studies. Knowledge on the vector control measures using larvicides (0.8–79.2%) [19, 31, 35] and biological measures (2.9–54.7%) [21, 23, 33] were the least known control measures. Whereas knowledge on using insecticides (5–83.2%) [19, 21, 22, 31, 32, 35] and “chemicals” (34–79.2%) [21, 32, 33], which is likely synonymous with insecticides were widely understood mosquito control measures.

**Table 4:** Knowledge of the general public on vector control

	Author (year)	Location	Sample size	Proportion	Preventative measure	Description of outcome
Source reduction of mosquito breeding areas						
[18]	Anand (2014)	India	100	47%	Source reduction	Knowledge on prevention of MBDs
[19]	Patil (2013)	India	154	12.5%	Source reduction	Knowledge on preventing chikungunya
[22]	Nagpal (2012)	India	1000	4–69% across study states	Source reduction	Knowledge on how to eliminate mosquito breeding
[45]	Aswathy (2011)	India	300	39%	Environmental sanitation	Knowledge of the means to prevent mosquito breeding
[33]	Boratne (2010)	India	1674	20.8%	Environmental	Knowledge on vector control measures
[21]	Arpit (2012)	India	274	34.7%	Environmental	Knowledge on mosquito control measures
[20]	Kohli (2013)	India	350	49.7%	Prevent stagnation of water	Knowledge on prevention of MBDs
[31]	Thakor (2015)	India	100	34.7%	Environmental	Knowledge on vector control measures
[32]	Mehta (2015)	India	120	31.6%	Regular cleaning of drainage	Knowledge of government measures to prevent MBDs
[32]	Mehta (2015)	India	103	66%	Keeping surroundings clean and proper drainage	Knowledge on prevention of MBDs
Source reduction from drinking water containers						
[18]	Anand (2014)	India	100	20%	Draining water and cleaning coolers	Knowledge on prevention of MBDs
[45]	Aswathy (2011)	India	300	20.7%	Overturning plastic cups, containers, and other receptacles	Knowledge of measures to prevent mosquito breeding
[20]	Kohli (2013)	India	350	41.1%	Cover water containers	Knowledge on prevention of MBDs
[20]	Kohli (2013)	India	350	21.1%	Cleaning of coolers	Knowledge on prevention of MBDs
[35]	Majra (2011)	India	150 cases, 450 non-cases	36% cases 64% non-cases	Changing stored water frequently	Knowledge on preventative measures
[35]	Majra (2011)	India	150 cases, 450 non-cases	40% cases 68% non-cases	Turning containers upside down	Knowledge on preventative measures
Biological						
[33]	Boratne (2010)	India	1674	2.9%	Biological	Knowledge on vector control measures
[23]	Ghosh (2011)	India	50	8%	Fish	Knowledge on mosquito control
[21]	Arpit (2012)	India	274	54.7%	Biological	Knowledge on mosquito control measures
[31]	Thakor (2015)	India	100	54.7%	Biological	Knowledge on vector control measures
Larvicides						
[19]	Patil (2013)	India	154	0.8%	Larvicides	Knowledge on preventing chikungunya
[35]	Majra (2011)	India	150 cases, 450 non-cases	4% cases 10% non-cases	Using abate	Knowledge on preventative measures
[31]	Thakor (2015)	India	100	79.2%	Anti-larval method	Knowledge on vector control measures
Insecticides						
[19]	Patil (2013)	India	154	33.1%	Insecticide spraying	Knowledge on preventing chikungunya
[22]	Nagpal (2012)	India	1000	5–48% across study States	Treatment with insecticides	Knowledge on how to eliminate mosquito breeding
[35]	Majra (2011)	India	150 cases, 450 non-cases	64% cases 68% non-cases	Spraying insecticides	Knowledge on preventative measures
[21]	Arpit (2012)	India	274	83.2%	Space spray	Knowledge on mosquito control measures
[31]	Thakor (2015)	India	100	83.2%	Space spray	Knowledge on vector control measures
[32]	Mehta (2015)	India	120	34.2%	Spraying and fogging	Knowledge of government measures to prevent MBDs
Unspecified Chemical						
[33]	Boratne (2010)	India	1674	61.1%	Chemical	Knowledge on vector control measures
[21]	Arpit (2012)	India	274	79.2%	Chemical	Knowledge on mosquito control measures
[32]	Mehta (2015)	India	103	34%	Spraying chemicals on water and keeping the surrounding clean	Knowledge on prevention of MBDs
[32]	Mehta (2015)	India	120	34.2%	Chemical spraying and cleaning of garbage	Knowledge of government measures to prevent MBDs
Other						
[43]	Raude (2009)	Mayotte	888	59.2%	Vector control	Knowledge of vector control
[33]	Boratne (2010)	India	1674	0.6%	Integrated	Knowledge on vector control measures
[20]	Kohli (2013)	India	350	38.6% 12%	Cleaning up garbage Putting kerosene oil in coolers	Knowledge on prevention of MBDs
[21]	Arpit (2012)	India	274	4%	Genetic method	Knowledge on mosquito control measures

*MBD* mosquito borne disease

Studies reported a large range in knowledge of personal protective measures (PPMs) (Table 5). Knowledge of the general public and health-care workers on the use of mosquito repellents and bed nets ranged from 0 to 92% in five studies from India [19, 21, 22, 32, 35], while knowledge of wearing protective clothing ranged (1–30%) across two studies from India [22, 45].

**Table 5:** Knowledge of the general public on personal protective measures (PPMs)

	Author (year)	Location	Sample size	Proportion	Preventative measure	Description of outcome
Mosquito repellents						
[21]	Arpit (2012)	India	274	53.3%	Repellents	Knowledge on personal control measures
[22]	Nagpal (2012)	India	1000	12–52% across study states	Repellents	Knowledge on how to protect yourself from mosquitoes
[35]	Majra (2011)	India	150 cases, 450 non-cases	72% cases 30% non-cases	Repellents	Knowledge on preventative measures
[31]	Thakor (2015)	India	100	53.3%	Repellents	Knowledge on personal protection
Mosquito nets						
[21]	Arpit (2012)	India	274	71.9%	Mosquito nets	Knowledge on personal control measures
[22]	Nagpal (2012)	India	1000	0–85% across study states	Bed nets	Knowledge on how to protect yourself from mosquitoes
[35]	Majra (2011)	India	150 cases, 450 non-cases	60% cases 92% non-cases	Mosquito nets	Knowledge on preventative measures
[31]	Thakor (2015)	India	100	71.9%	Mosquito nets	Knowledge on personal protection
Mosquito nets and repellents						
[19]	Patil (2013)	India	154	23.2%	Mosquito nets and repellents	Knowledge on preventing chikungunya
Protective clothing						
[22]	Nagpal (2012)	India	1000	1–30% across study states	Wear body covering clothing	Knowledge on how to protect yourself from mosquitoes
[35]	Majra (2011)	India	150 cases, 450 non-cases	7% cases 24% non-cases	Wearing full dresses	Knowledge on preventative measures
Mosquito proofing home						
[22]	Nagpal (2012)	India	1000	0–6% across study states	Make house mosquito proof	Knowledge on how to protect yourself from mosquitoes
[35]	Majra (2011)	India	150 cases, 450 non-cases	30% cases 70% non-cases	Screening of houses	Knowledge on preventative measures
Other						
[20]	Kohli (2013)	India	350	5.7%	Using PPMs	Knowledge on prevention of MBDs
[43]	Raude (2009)	Mayotte	888	60.5%	Self-protective behavior	Knowledge of self-protective behavior
[18]	Anand (2014)	India	100	93%	PPMs	Knowledge on prevention of MBDs
[46]	Vaidya (2013)	India	178	74.2%	Cleaning	Knowledge on protective measures

*MBD* mosquito borne disease

When a group of 1462 students in Nicaragua were surveyed about the effectiveness of several preventative measures against mosquito bites, the most commonly reported measures were bed nets (60.6%), sprays (60.5%), window nets (58.6%), and the removal of stagnant water from containers (58.5%) [29]. Wearing protective clothing (35%) and closing windows (33%) were perceived by this group to be less effective at reducing mosquito bites [29].

Knowledge on the availability of a vaccine for chikungunya was assessed in two studies [26, 29]. In a group of students from French Guiana (*n* = 1462), 16% believed there was a vaccine for chikungunya [29]. Of the community service volunteers from the USA traveling to the Dominican Republic who had self-reported pre-travel knowledge on chikungunya, 67% (*n* = 19) correctly answered there is no vaccine available for chikungunya [26]. A community opinion survey on the use of genetically modified mosquitoes as a method of mosquito management was conducted in the USA [44]. This novel approach to mosquito control was opposed by 58% of the respondents (*n* = 86) and the remaining 42% were either neutral or supported the method [44]. When multiple vector control measures were ranked on a scale of 1–5 (1 being the method they support the most and 5 the least), this group was most supportive of draining water on private property to reduce mosquito breeding (mean = 1.98) and least supportive of genetically modified mosquitoes to reduce the mosquito population (mean = 4.14) [44].

### Qualitative studies

Two qualitative research articles were identified from the same author [50, 53], both focusing on local experiences and responses to the 2005–2007 chikungunya epidemic in La Réunion. The first article reported on an anthropological study consisting of interviews with 16 residents who believed they had been ill with the disease [53]. Participants had differing beliefs regarding the etiology of chikungunya, with seven participants leaning toward biomedical explanations of CHIKV as vector-borne, while others thought that the infection was air-borne or due to poor sanitary conditions [53].

“Although *bann la* [they, all of them] said that chikungunya is a mosquito, I don’t think so because mosquitoes have always existed here in Réunion” (quote from participant, reported in Jansen, 2012).

The second study reported on a discourse analysis of 111 local newspaper articles published in La Réunion during the chikungunya epidemic [50]. These newspapers functioned as a secondary source to communicate local perceptions and experiences with the epidemic [50]. The analysis revealed that coverage of the epidemic began as informational, but became increasingly political at the height of the epidemic, with criticisms of the government’s response [50]. For instance, many Réunionese did not believe that chikungunya was transmitted by mosquitoes, but rather that the state was keeping important information from them [50].

## Discussion

Risk perceptions, attitudes, and knowledge of chikungunya among the public and health professionals vary across populations and countries as shown in this systematic review. The results suggest that the majority of the populations in the captured studies are uncertain, unaware, or do not understand chikungunya and/or mosquito borne diseases. This is a potential barrier to community and personal protective actions.

Based on the studies captured in this review, there has been limited research on this subject. By employing the scoping and systematic review methodologies and including studies in multiple languages (English, French, Spanish, and Portuguese), we attempted to identify and include all the relevant research on this topic. However, it is possible that some non-indexed studies or studies in other languages were missed. Many of the identified studies investigated participants’ knowledge, perceptions, and attitudes of general mosquito borne diseases rather than chikungunya specifically. Also, in many of the countries where people are at risk of contracting chikungunya, they are also at risk of contracting malaria or dengue, which tend to be more well-known diseases among the general public [18]. This may explain why some populations were knowledgeable on vector control and personal protective measures but have low levels of awareness on chikungunya. Although the disease is endemic in many areas of Africa, Asia, and the Pacific and Indian Ocean Islands, the majority of the studies to date (43%, *n* = 37) were conducted in India which has recorded outbreaks of chikungunya for several decades, or La Réunion Island, which experienced a large outbreak in 2005–2006. Based on the geographic distribution of chikungunya, the current studies do not represent all chikungunya-affected populations or even a representative sample. Thus, the knowledge, attitudes, and perceptions of chikungunya-affected populations that are not represented may be different and their absence is a knowledge gap in this review.

The risk of bias assessment and data extraction revealed that the studies captured in this review were not well reported and were missing some critical details. This decreases our confidence in the findings of the studies identified and prevents a reliable interpretation of their results. Across studies, the outcomes reported were not comparable to each other thus preventing synthesis of results or comparisons across populations. Future standardization of outcomes would improve the comparability of results across studies making them easier to synthesize and draw more generalizable conclusions about the consistency, direction, and magnitude of the results.

There was a lack of qualitative research on the topic, and it was suggested that there is a need for greater understanding and consideration of varying cultural explanations and for conceptualizations of CHIKV etiology to more effectively address and respond to outbreaks in affected communities [53]. This is supported by some of the beliefs and misunderstandings of respondents in several surveys from this systematic review. The importance of cultural practices and community perception can be critical to the success of epidemic control measures as was the case during the 2014 Ebola outbreak in West Africa [54]. Social stigmatization and deeply ingrained cultural practices such as ritual washing of the deceased and the consumption of bush meat threatened the success of mitigation efforts [55, 56]. Investigating underlying social factors associated with chikungunya and barriers and facilitators to potential mitigation options through qualitative research would be useful for designing future education and control strategies.

Risk perceptions, attitudes, beliefs, and knowledge are important predictors of an individuals’ behavior toward mosquito borne disease [57, 58]. The relationship between these variables and an individuals’ behavior can be explained and predicted by different theories of behavior change, such as the Health Belief Model, Theory of Planned Behavior, and the Stages of Change Model [59]. These theories provide formal and structured frameworks for investigating predictors of health behaviors and for designing health behavior change inventions; however, only nine studies in this review used a formal theory of behavior change to guide their data collection [22, 25, 29, 34, 36, 40, 45, 46, 60]. Future studies should use theories of behavior change to investigate the psychosocial risk factors for chikungunya prevention and to design future education and control strategies for CHIKV.

Studies that looked at the general knowledge and perceptions on chikungunya varied across populations and countries. As expected, higher awareness of chikungunya was found in areas affected by an outbreak. This is most likely due to public education, media, and personal experience with the disease. There may also be a difference between recognizing a disease with no depth in knowledge, which is perceived as being informed, compared to factual knowledge about the disease. One study identified literacy, a socio-economic factor that can be considered a surrogate for education and knowledge, as a significant confounder [33]. Other studies found that gender and age were predictors of higher knowledge levels; however, these findings were less consistent and should be investigated further.

Limited studies were conducted on the perceptions of the risk and severity of chikungunya. Two studies that found a high perceived severity of the disease were both conducted on the Indian Ocean Islands [41, 43]. This is to be expected as the studies occurred shortly after the large outbreak in 2005–2006. In contrast, the study in France, where many mosquito borne diseases are not endemic, showed that although the participants recognized the severity of mosquito borne diseases, they were not worried about contracting them [30].

There was a lot of heterogeneity in the level of knowledge on transmission of CHIKV by mosquitoes between studies, likely due to the outbreak status of the area and the amount of public education that had occurred. Knowledge on general mosquito breeding sites was generally higher, which could be attributed in part to the fact that malaria and dengue are endemic in many of these countries and are well-known diseases among the general public. Higher knowledge about vector control and personal protective measures in endemic countries such as India are also likely the result of the presence of many endemic mosquito borne diseases.

The perception of personal control or “self-responsibility” for mitigation was only investigated in a few studies. Since self-efficacy is one of the most important variables in most of the theories of behavior change [59], it would be useful to study perception of control over protection from CHIKV or mosquito borne diseases as a determinant of personal protective behaviors and what education strategies are most likely to empower the individual to participate in proposed mitigation strategies.

This review encompassed knowledge, attitudes, and perceptions of both the general public and health professionals. However, the majority (86.5%, *n* = 37) of studies were conducted on the general public. There were no direct comparisons done between the general public and health professionals. Only one outcome, knowledge of CHIKV transmission by mosquitoes, looked at both populations. As expected, health professionals had higher levels of knowledge that mosquitoes transmit CHIKV than the general public. Most of the studies with health professionals measured knowledge of symptoms and perceptions on various treatments. Information from different populations is needed to inform the design of future education and control strategies.

Almost all populations in the studies included in this review were from developing countries with a large proportion of poorly educated individuals that have little to no disposable income. Thus, the affordability of mitigation measures needs to be considered when developing control strategies. For example, although it was shown that insecticides were the most commonly known mitigation strategy, that might not be the best strategy for a community with no disposable income for the insecticides.

## Conclusion

Overall, this review identified, assessed, and analyzed the global literature on the knowledge, attitudes, and perceptions of CHIKV and its transmission in affected populations. The results indicated that there is variability across populations and countries, but most of the captured populations are uncertain, unaware, or do not understand chikungunya and/or mosquito borne diseases. As the disease continues to spread into previously non-endemic areas, it is recommended that research efforts be increased to close some of the knowledge gaps or better understand the uncertainty identified in this SR with respect to the impact knowledge, attitudes, and perceptions of chikungunya and personal protective measures can have on affected and non-affected populations. Investigations into what motivates individuals to adopt personal protective and vector control measures at home and within their communities will aid in the design and implementation of effective education and control strategies. Researchers in this area are encouraged to follow guidelines on conduct and reporting based on study design to minimize bias in their research and enhance the clarity of their article for use by other researchers and decision makers.

## Additional files

Additional file 1: Protocol for SR of the risk perceptions, attitudes, and/or knowledge of chikungunya among the public and health professionals (DOCX 73 kb)

Additional file 2: Citation list of relevant articles (DOCX 41 kb)

Additional file 3: PRISMA flow diagram of articles through the scoping review process (personal communication M. Mascarenhas 2017) (DOCX 27 kb)

## Abbreviations

CHIKV: Chikungunya virus
CINAHL: The Cumulative Index to Nursing and Allied Health Literature
LILACS: Latin American and Caribbean Health Sciences Literature
MBD: Mosquito borne disease
PPM: Personal protective measure
PRISMA: Preferred Reporting Items for Systematic Reviews and Meta-Analyses
SR: Systematic review
