# Supplementary material for: Risk perceptions, attitudes, and knowledge of chikungunya among the public and health professionals: a systematic review
Source: Trop Med Health. 2017 Sep 4;45:21. doi: 10.1186/s41182-017-0061-x (PMC5582396; doi:10.1186/s41182-017-0061-x)
Supplement: Supplementary file 2 — Citation list of relevant articles (DOCX 41 kb) [file 41182_2017_61_MOESM2_ESM.docx]

### Additional file 2: Citation list of relevant articles

## References:

Abramides, G. C., Roiz, D., Guitart, R., Quintana, S., & Gimenez, N. (2013). Control of the Asian tiger mosquito (Aedes albopictus) in a firmly established area in Spain: risk factors and people’s involvement. *Royal Society of Tropical Medicine and Hygiene, 107*(11), 706-714. doi:10.1093/trstmh/trt093

Adalja, A., Sell, T. K., McGinty, M., & Boddie, C. (2016). Genetically Modified (GM) Mosquito Use to Reduce Mosquito-Transmitted Disease in the US: A Community Opinion Survey. *PLOS Current Outbreaks, 1*. doi:10.1371/currents.outbreaks.1c39ec05a743d41ee39391ed0f2ed8d3

Anand, T., Kumar, R., Saini, V., Meena, G., & Ingle, G. (2014). Knowledge and Use of Personal Protective Measures Against Mosquito Borne Diseases in a Resettlement Colony of Delhi. *Annals of Medical and Health Sciences Research, 4*(2), 227-232. doi:10.4103/2141-9248.129048

Arpit, P., Sonal, P., Manish, F., & DV, B. (2012). Impact of Educational Intervention Regarding Mosquito Borne Diseases and Their Control Measures among The Link Workers of Urban Health Centers (UHCs) of Ahmedabad City. *National Journal of Community Medicine, 3*(2), 178-182.

Aswathy, S., Dinesh, S., Beteena, K., Johnson, A., & Leelamoni, K. (2011). A Post-Epidemic Study on Awareness of Vector Habits of Chikungunya and Vector Indices in A Rural Area of Kerala. *Journal of Communicable Diseases, 43*(3), 209-215.

Bedoya-Arias, J. E., Murillo-García, D. R., Bolaños-Muñoz, E., Hurtado-Hurtado, N., Ramírez-Jaramillo, V., Granados-Álvarez, S., & Rodríguez-Morales, A. J. (2015). Healthcare students and workers' knowledge about epidemiology and symptoms of chikungunya fever in two cities in Colombia. *The Journal of Infection in Developing Countries, 9*(3), 330-332. doi:10.3855/jidc.6445

Boratne, A., Jayanthi, V., Datta, S., Singh, Z., Senthilvel, V., & Joice, Y. (2010). Predictors of knowledge of selected mosquito-borne diseases among adults of selected peri-urban areas of Puducherry. *Journal of Vector Borne Diseases, 47*(4), 249-256.

Boyer, S., Foray, C., & Dehecq, J.-S. (2014). Spatial and Temporal Heterogeneities of Aedes albopictus Denisty in La Reunion Island: Rise and Weakness of Entomological Indices. *PLoS ONE, 9*(3), 1-12. doi:10.1371/journal.pone.0091170.

Cherry, C. C., Beer, K. D., Fulton, C., Wong, D., Buttke, D., Staples, J. E., & Ellis, E. M. (2016). Knowledge and use of prevention measures for chikungunya virus among visitors - Virgin Islands National Park, 2015. *Travel Medicine and Infectious Disease, 14*(5), 475-480. doi:10.1016/j.tmaid.2016.08.011

Claeys, C., Robles, C., Bertaudiere-Montes, V., Deschamps-Cottin, M., Megnifo, H. T., Pelagie-Moutenda, R., . . . Fouque, F. (2016). Socio-ecological factors contributing to the exposure of human populations to mosquito bites that transmit dengue fever, chikungunya and zika viruses: a comparison between mainland France and the French Antilles. *Environnement, Risques & Santé, 15*(4), 318-325. doi:10.1684/ers.2016.0870

Dilip, C., Saraswathi, R., Krishnan, P., Azeem, A., Raseena, azeez, A., . . . jose, J. (2010). Comparative evaluation of different systems of medicines and the present scenario of chikungunya in Kerala. *Asian Pacific Journal of Tropical Medicine*, 443-447.

Doke, P. P., Satvaji-Dakhure, D., & Patil, A. V. (2011). A clinico-epidemiological study of chikungunya outbreak in Maharashtra state, India. *Indian Journal of Public Health, 55*(4), 313-316. doi:10.4103/0019-557X.92413

Fenetrier, E., Sissoko, D., Vernazza-Licht, N., Bley, D., Gaiizcre, B.-A., & Malvy, D. (2013). Feedback from primary care practitioners two years after the chikungunya epidemic on Reunion. *Bull. Soc. Pathol. Exot., 106*, 193-200. doi:DOI I0.1007/s13149-013-0295-8

Flahault, A., Aumont, G., Boisson, V., de Lamballerie, X., Favier, F., Fontenille, D., . . . Gaüzere, B. (2012). An interdisciplinary approach to controlling chikungunya outbreaks on french islands in the south-west indian ocean. *Médecine Tropicale, 72*, 66-71.

Fritzell, C., Raude, J., Adde, A., Dusfour, I., Quenel, P., & Flamand, C. (2016). Knowledge, Attitude and Practices of Vector-Borne Disease Prevention during the Emergence of a New Arbovirus: Implications for the Control of Chikungunya Virus in French Guiana. *PLOS Neglected Tropical Diseases, 10*(11), e0005081. doi:10.1371/journal/pntd.000501

Ghosh, S. K., Chakaravarthy, P., Panch, S. R., Krishnappa, P., Tiwari, S., Ojha, V. P., . . . Dash, A. P. (2011). Comparative efficacy of two poeciliid fish in indoor cement tanks against chikungunya vector Aedes aegypti in villages in Karnataka, India. *BMC Public Health, 11*, 599. doi:10.1186/1471-2458-11-599

Goorah, S., Dewkurun, M. K., & Ramchurn, S. K. (2013). Assessing the sustainability of individual behavior change against mosquitos after the outbreak of a vector-borne disease in Mauritius: a case study. *Internet Journal of Medical Update, 8*(1), 9-16.

Hsu, L., Jin, J., Ang, B., Kurup, A., & Tambyah , P. (2011). Hand hygiene and infection control survey pre- and peri-H1N1-2009 pandemic: knowledge and perceptions of final year medical students in Singapore. *Singapore Medical Journal, 52*(7), 486-490.

Jansen, K. A. (2012). The printed press's representations of the 2005-2007 chikungunya epidemic Réunion: Political polemics and (post)colonial disease. *Journal of African Media Studies, 4*(2), 227-242. doi:10.1386/jams.4.2.227_1

Jansen, K. A. (2013). The 2005-2007 Chikungunya Epidemic in Reunion: Ambiguous Etiologies, Memories, and Meaning-Making. *Medical Anthropology, 32*(2), 174-189. doi:10.1080/01459740.2012.679981

Kohli, C., Kumar, R., Meena, G., Singh, M., & Ingle, G. (2013). Awareness about Mosquito Borne Diseases in Rural and Urban Areas of Delhi. *Journal of Communicable Diseases, 45*(3&4), 201-207.

Kuan, G., Ramirez, S., Gresh, L., Ojeda, S., Melendez, M., Sanchez, N., . . . Harris, E. (2016). Seroprevalence of Anti-Chikungunya Virus Antibodies in Children and Adults in Managua, Nicaragua, After the First Chikungunya Epidemic, 2014-2015. *PLOS Neglected Tropical Diseases, 10*(6), e0004773. doi:10.1371/journal.pntd.0004773

Marja, J. P., & Acharya, D. (2011). Impact of knowledge and practices on prevention of chikungunya in an epidemic area in India. *Annals of Tropical Medicine and Public Health, 4*(1), 3-6.

Mehta, D., Solanki, H., Patel, P., Umat, P., Chauhan, R., Shukla, S., & Singh, M. (2015). A Study on Knowledge, Attitude & Practice Regarding Mosquito Borne Diseases in an Urban Area of Bhavnagar. *Healthline Journal, 6*(2), 29-33.

Millman, A. J., Esposito, D. H., Biggs, H. M., Decenteceo, M., Klevos, A., Hunsperger, E., . . . Jentes, E. S. (2016). Chikungunya and Dengue Virus Infections among United States Community Service Volunteers Returing from the Dominican Republic, 2014. *The American Journal of Tropical Medicine and Hygiene, 94*(6), 1336-1341. doi:10.4269/ajtmh.15-0815

Moro, M., Gagliotti, C., Silvi, G., Angelini, R., Sambri, V., Rezza, G., . . . Macini, P. (2010). Knowledge, attitudes and practices survey after an outbreak of chikungunya infections. *International Health, 2*, 223-227. doi:10.1016/j.inhe.2010.07.003

Nagpal, B., Saxena, R., Srivastava, A., Singh, N., Ghosh, S., Sharma, S., . . . Dash, A. (2012). Retrospective study of chikungunya outbreak in urban areas of India. *The Indian Journal of Medical Research, 135*(3), 351-358.

Patil, S. S., Patil, S. R., Durgawale, P., & Patil, A. (2013). A study of the outbreak of Chikungunya fever. *Journal of Clinical and Diagnostic Research, 7*(6), 1059-1062. doi:10.7860/JCDR/2013/5330.3061

Puwar, T., Shetha, J. K., Kohli, V., & Yadav, R. (2010). Prevalence of chikungunya in the city of Ahmedabad, India, during the 2006 outbreak:. *WHO Dengue Bulletin*, pp. 40-45. Retrieved from http://www.who.int/iris/handle/10665/170980

Raude, J., & Setbon. (2009). The role of environmental and individual factors in the social epidemiology of chikungunya disease on Mayotte Island. *Health & Place, 15*, 689-699. doi:10.1016/j.healthplace.2008.10.009

Raude, J., Chinfatt, K., Huang, P., Betansedi, C. O., Katumba, K., Vernazza, N., & Bley, D. (2012). Public perceptions and behaviours related to the risk of infection with Aedes mosquito-borne diseases: a cross-sectional study in Southeastern France. *BMJ Open, 2*(6). doi:10.1136/bmjopen-2012-002094

Setbon, M., Raude, J., & Pottratz, D. (2008). Chikungunya on Réunion Island: Social, Environmental and Behavioural Factors in an Epidemic Context. *Population, 63*(3), 491-518.

Surendran, S. N., Kannathasan, S., Kajatheepan, A., & Jude, P. J. (2007). Chikungunya-type fever outbreak: some aspects related to this new epidemic in Jaffna district, northern Sri Lanka. *Tropical Medicine and Health, 35*(3), 249-252.

Tenglikar, P. V., Hussain, M., Nigudgi, S., & Ghooli, S. (2016). Knowledge and practices regarding mosquito borne disease among people of an urban area in Kalaburgi, Karnataka. *National Journal of Community Medicine, 7*(3), 223-225.

Thakor, N. C., Vikani, S. K., & Nagar, A. A. (2015). Impact of educational intervention regarding mosquito-borne diseases and their control measures among multipurpose health workers (MPHWs) of Patan district, Gujarat, India. *International Journal of Medical Science and Public Health, 4*(11), 1620-1623. doi:10.5455/ijmsph.2015.01052015333

Thuilliez, J., Bellia, C., Dehecq, J.-S., & Reilhes, O. (2014). Household-Level Expenditure on Protective Measures Againts Mosquitoes on the Island of La Reunion, France. *PLoS Neglected Tropical Diseases, 8*(1), e2609. doi:10.1371/journal.pntd.0002609

Vaidya, V., & Sawant, S. (2013). A KAP Study in Pune City Involving School Children as a Strategy for effective Vector Control in Chikungunya. *Indian Journal of Public Health Research & Development, 4*(4). doi:10.5958/i.0976-5506.4.4.181
