# Supplementary material for: Risk perceptions, attitudes, and knowledge of chikungunya among the public and health professionals: a systematic review
Source: Trop Med Health. 2017 Sep 4;45:21. doi: 10.1186/s41182-017-0061-x (PMC5582396; doi:10.1186/s41182-017-0061-x)
Supplement: Supplementary file 3 — PRISMA flow diagram of articles through the scoping review process (personal communication M. Mascarenhas 2017) (DOCX 27 kb) [file 41182_2017_61_MOESM3_ESM.docx]

Additional file 3: PRISMA flow diagram of articles through the scoping review process (personal communication M. Mascarenhas, 2017)

Search

Relevance screening

Data extraction

**Search total**

N =17,445

**Relevance Screening 1**

N = 6,820

**Relevance Screening 2**

N = 3,427

**Articles screened**

N = 2,374

**Excluded** (duplicates)

N = 10,625

**Excluded** (not relevant)

N = 3,393

**Excluded** (not relevant)

N = 1,053

**Excluded** (not relevant)

N = 453
